# The localization of NADPH oxidase and reactive oxygen species in in vitro-cultured *Mesembryanthemum crystallinum* L. hypocotyls discloses their differing roles in rhizogenesis

**DOI:** 10.1007/s00709-014-0692-2

**Published:** 2014-08-30

**Authors:** Marta Libik-Konieczny, Małgorzata Kozieradzka-Kiszkurno, Christine Desel, Żaneta Michalec-Warzecha, Zbigniew Miszalski, Robert Konieczny

**Affiliations:** 1 The Franciszek Górski Institute of Plant Physiology, Polish Academy of Sciences, Niezapominajek 21 St., 30-239 Kraków, Poland; 2 Department of Plant Cytology and Embryology, University of Gdańsk, Wita Stwosza 59 St., 80-308 Gdańsk, Poland; 3 Institute of Botany, Christian-Albrechts-University of Kiel, Olshausenstr. 40, 24098 Kiel, Germany; 4 Malopolska Centre of Biotechnology, Jagiellonian University, Gronostajowa 7A St., 30-387 Kraków, Poland; 5 Department of Plant Cytology and Embryology, Institute of Botany, Jagiellonian University, Gronostajowa 9 St., 30-387 Kraków, Poland

**Keywords:** Adventitious root, Ice plant, Organogenesis, Oxidative stress, Tissue culture, Transmission electron microscopy

## Abstract

This work demonstrated how reactive oxygen species (ROS) are involved in the regulation of rhizogenesis from hypocotyls of *Mesembryanthemum crystallinum* L. cultured on a medium containing 1-naphthaleneacetic acid (NAA). The increase of NADPH oxidase activity was correlated with an increase of hydrogen peroxide (H_2_O_2_) content and induction of mitotic activity in vascular cylinder cells, leading to root formation from cultured hypocotyls. Diphenylene iodonium (DPI), an inhibitor of NADPH oxidase, inhibited H_2_O_2_ production and blocked rhizogenesis. Ultrastructural studies revealed differences in H_2_O_2_ localization between the vascular cylinder cells and cortex parenchyma cells of cultured explants. We suggest that NADPH oxidase is responsible for H_2_O_2_ level regulation in vascular cylinder cells, while peroxidase (POD) participates in H_2_O_2_ level regulation in cortex cells. Blue formazan (NBT) precipitates indicating superoxide radical (O_2_^•−^) accumulation were localized within the vascular cylinder cells during the early stages of rhizogenesis and at the tip of root primordia, as well as in the distal and middle parts of newly formed organs. 3,3′-diaminobenzidine (DAB) staining of H_2_O_2_ was more intense in vascular bundle cells and in cortex cells. In newly formed roots, H_2_O_2_ was localized in vascular tissue. Adding DPI to the medium led to a decrease in the intensity of NBT and DAB staining in cultured explants. Accumulation of O_2_^•−^ was then limited to epidermis cells, while H_2_O_2_ was accumulated only in vascular tissue. These results indicate that O_2_^•−^ is engaged in processes of rhizogenesis induction involving division of competent cells, while H_2_O_2_ is engaged in developmental processes mainly involving cell growth.

## Introduction

Processes of organ development in vitro require the re-initiation of cell division and the modulation of cell differentiation in cultured explants. Organogenesis is composed of three sequential phases (Christianson and Warnick 1983, 1984, 1985; Sugiyama 1999; Zhang and Lemaux 2004; Zhao et al. 2008). During the first phase, explant cells acquire organogenic competence, the ability to recognize signals that commit them to a particular developmental program. During the second phase, already competent but still quiescent cells re-enter the cell cycle, proliferate, and alter their developmental fate, the key step during de novo organogenesis. The last phase of organogenesis is cell differentiation and organ development. Particular developmental fates in in vitro culture have been shown to be largely controlled by the balance of cytokinin and auxin (Skoog and Miller 1957; Zhao et al. 2008). As with many other plant species, in *Mesembryanthemum crystallinum* hypocotyl culture, auxin alone or in combination with a low level of cytokinin promotes root primordial formation, but shoots are induced when the cytokinin concentration exceeds that of auxin (Konieczny et al. 2009). This hormonal signaling interaction is not the only means of controlling the transition from proliferation to differentiation. Stresses (osmotic shock, culture medium dehydration, water stress, heavy metal ions, changes in culture medium pH, heat or cold shock, hypoxia, antibiotics, UV radiation, mechanical stress) that lead to overproduction of reactive oxygen species (ROS) have been mentioned as factors inducing signals for organogenesis (Potters et al. 2007).

The reactive oxygen species include a variety of reactive oxidants: superoxide radical (O_2_^•−^), hydroxyl radical (OH˙), hydrogen peroxide (H_2_O_2_), and others. They can play an important role in many biological processes as integrated signaling molecules functioning together with many other signaling networks (Apel and Hirt 2004; Mittler et al. 2011). Integration of stress-elicited overproduced ROS with the auxin signaling network leads to stress-induced morphogenic responses (Potters et al. 2007).

ROS are generated intracellularly by several organelles including the mitochondria, chloroplasts, and peroxisomes, but the primary source of ROS involved in signaling cascades and plant development is the apoplast, where they are produced mainly by a membrane-bound enzyme, NADPH oxidase (NOX). This enzymatic complex, in plants also known as a respiratory burst oxidase homologue (RBOH), generates O_2_^•−^ by transferring electrons from NADPH to molecular oxygen (Gapper and Dolan 2006). The highly reactive O_2_^•−^ undergoes dismutation to form H_2_O_2_ either spontaneously or via the superoxide dismutase (SOD) enzyme (Mori and Schroeder 2004). H_2_O_2_ is less reactive than O_2_^•−^_,_ but it is more stable and can diffuse through membranes via aquaporins (Bienert et al. 2007). Thus, H_2_O_2_ is recognized as the most potent signaling ROS in plants. Among other functions, it participates in the regulation of cell state decisions and influences the induction of proliferation and differentiation (Neill et al. 2002; Dunand et al. 2007; Sarsour et al. 2008; van Breusegem et al. 2008; Owusu-Ansah and Banerjee 2009; Tsukagoshi et al. 2010). Proteomic analysis has shown that H_2_O_2_-induced proteins are related to plant signaling during cell elongation and division (Barba-Espin et al. 2010). Results from transcriptome analysis (Gadjev et al. 2006) demonstrated that changes in ROS level lead to a clear reorientation of the plant’s genome transcription and also produced experimental evidence for the specific signaling capacities of different ROS and for the importance of their subcellular localization in the plant cell. Their biological properties (e.g., chemical reactivity, half-life, lipid solubility) differ between particular ROS (D’Autréaux and Toledano 2007).

Organogenesis is a complex morphogenetic phenomenon requiring massive reorganization of genome transcription. Some of the elementary processes and essential genes involved in this composite phenomenon have been identified (Sugiyama 1999; Philips 2004; Su et al. 2007), but the regulation of their transcriptional activities underlying the processes of cell regeneration is poorly understood. The regulatory function of ROS and the antioxidant system in morphogenesis in vitro has been studied extensively (Siminis et al. 1993; de Marco and Roubelakis-Angelakis 1996; Kairong et al. 1999; Papadakis and Roubelakis-Angelakis 2002; Libik et al. 2005; Li et al. 2007, 2009a, b; Baťková et al. 2008; Konieczny et al. 2008; Gupta 2010; Vatankham et al. 2010; Agrawall and Purohit 2012; Libik-Konieczny et al. 2012). In vitro culture systems are good models for studying the physiological events in organogenesis since they make it easy to harvest a large amount of synchronized culture samples showing the state of organogenesis.

In recent studies of *M. crystallinum* hypocotyls during successive stages of rhizogenesis on a medium containing 2,4-dichlorophenoxy-acetic acid (2,4-D), explants became competent to respond to the rhizogenic action of auxin on day 3 of culture, when the hydrogen peroxide content of cultured tissue reached high levels and the activity of the manganese form of superoxide dismutase, MnSOD-2, was induced (Konieczny et al. 2014). Here, we assess the concentration and distribution of ROS versus the activity of NADPH oxidase during successive stages of rhizogenesis induced on a medium containing 1-naphthaleneacetic acid (NAA). Studies on O_2_^•−^ and H_2_O_2_ localization should shed light on how different ROS compounds can be used to elicit regeneration from competent cells of explants.

## Materials and methods

### Plant material

*M. crystallinum* L. (common ice plant) seeds were obtained from plants growing in the greenhouse. The seeds were surface-sterilized by immersion in 70 % (*v*/*v*) ethanol for 2 min, then in a commercial bleach solution diluted with water (1:2; *v*/*v*) for 10 min, and finally in a more diluted bleach solution (1:10; *v*/*v*) for 5 min. Following sterilization, the *M. crystallinum* seeds were rinsed three times with sterile, distilled water and then placed onto 9-cm Petri dishes (30–50 seeds per dish) containing 20 ml of a solid medium consisted of basal Murashige and Skoog (1962) salts and vitamins (Sigma-Aldrich, Poland), 30 g l^−1^ sucrose, and 7 g l^−1^ agar (Difco Bacto, USA). The dishes with seeds were placed in a growth chamber for germination at 25/20 °C under a 16/8-h light/dark photoperiod with light provided by cool fluorescent light, 150–200 mmol m^−2^ s^−1^. After 10 days of germination, the hypocotyls (5–7 mm in length) were excised from the seedlings and placed on culture media.

### Induction of rhizogenesis in in vitro culture conditions

To induce rhizogenesis, the explants were placed horizontally on a root-inducing medium (RIM) composed of a solid MS basal medium supplemented with 1 mg l^−1^ 1-naphthaleneacetic acid (Sigma-Aldrich, Poland), pH 5.7. Some explants were put on this medium supplemented with 31.4 mg l^−1^ diphenylene iodonium (DPI, Sigma-Aldrich, Poland)—an irreversible inhibitor of flavin adenine dinucleotide (FAD) cofactor containing enzymes such as NOX (Gapper and Dolan 2006). Hypocotyls excised from *M. crystallinum* seedlings (day 0) and cultured for 3, 5, 7, and 10 days on RIM and RIM + DPI were used as experimental material. For each analysis, 30 hypocotyls were collected from three Petri dishes containing ten hypocotyls each.

### Histological studies

The material for observation in light microscopy was fixed and embedded following procedures described earlier (Konieczny et al. 2012). Sections (5 μm in thickness) were stained with 0.1 % (*v*/*w*) aquatic solution of toluidine blue O (Sigma-Aldrich, Poland) according to O’Brien et al. 1964. The dye binds to nucleic acids and acidic proteins allowing to visualize structural details of examined tissue.

### Quantification of H_2_O_2_ concentration

Endogenous H_2_O_2_ levels were determined according to the method described previously by Brennan and Frenkel (1977), with some modifications. H_2_O_2_ was extracted by homogenization of 0.5–1 g tissue in 2 ml cold acetone. After centrifugation (5 min at 12,000*g*), the pellet was discarded and a 0.5 ml extract was collected. A titanium reagent (50 μl 20 % titanium tetrachloride in concentrated HCl, *v*/*v*, Sigma-Aldrich, Poland) was added to the 0.5 ml extract, followed by the addition of 0.1 ml NH_3_ (25 %; *v*/*v*) to precipitate the peroxide-titanium complex. After 5 min centrifugation at 10,000*g*, the supernatant was discarded and the precipitate was repeatedly washed in 1 ml acetone and centrifuged again for 5 min at 10,000*g*. The precipitate was solubilized in 1 ml 1N H_2_SO_4_ and brought to a final volume of 2 ml. The absorbance of the obtained solution was read at 415 nm against a water blank. The concentration of peroxide in the extract was determined by comparing absorbance against a standard curve representing the titanium–H_2_O_2_ complex over a range from 0 to 20 μmol ml^−1^. All H_2_O_2_ measurements were normalized to tissue fresh weight.

### Electron microscopy studies of H_2_O_2_ localisation

Hydrogen peroxide ultrastructural localization was assessed via determination of cerium perhydroxide (Ce(OH)_2_OOH and Ce(OH)_3_OOH) formation after the reaction of cerium chloride (CeCl_3_, Sigma-Aldrich, Poland) with endogenous H_2_O_2_ which gives rise to electron-dense precipitates detectable by transmission electron microscopy (Bestwick et al. 1997). Small tissue samples were preincubated for 1 h in a 50 mmol l^−1^ morpholinepropanesulfonic acid (MOPS, Sigma-Aldrich, Poland) buffer (pH 7.0), containing 5 mmol l^−1^ CeCl_3_. Subsequently, tissues were quickly washed in the buffer and fixed in 2 % formaldehyde (prepared from paraformaldehyde) and a 2.5 % (*v*/*v*) glutaraldehyde in 0.1 mol l^−1^ cacodylate buffer (pH 7.0) for 4 h at room temperature. The procedure for preparing the samples for transmission electron microscopy was as described earlier (Kozieradzka-Kiszkurno et al. 2012). The material was dehydrated in a series of graded acetone and embedded in Spurr Low-Viscosity Embedding Kit (Polysciences, Germany). Ultrathin (60–100 nm) sections were cut with a diamond knife on a Sorvall MT 2B ultramicrotome and transferred to 200 mesh grids. The sections were stained with uranyl acetate and lead citrate and then viewed using a Philips CM 100 transmission electron microscope.

The controls were tissue samples preincubated for 15 min in 10 mmol l^−1^ sodium pyruvate, a strong H_2_O_2_ scavenger (Li et al. 1998).

### Measurements of NOX activity

Hypocotyls were homogenized in a protein extraction buffer containing 0.25 mol l^−1^ sucrose, 10 mmol l^−1^ Tris–HCl, 1 mmol l^−1^ ethylenediaminetetraacetic acid (EDTA, Sigma-Aldrich, Poland), and 2.5 mmol l^−1^ dithiothreitol (DTT, Sigma-Aldrich, Poland), pH 7.2, and filtered through a cheesecloth. The filtrate was centrifuged at 4 °C at 10,000*g* for 15 min. The supernatant was transferred to a new tube and used directly to extract membranes. The membrane fraction was separated from the supernatant by centrifugation at 80,000*g* for 30 min, according to the procedure described by Janeczko et al. (2008). The pellet was then resuspended in a Tris–HCl dilution buffer and used immediately for further analysis. The protein content in the isolated membrane fraction was determined according to the method of Bradford (1976) with albumin from bovine serum albumin (BSA, Sigma-Aldrich, Poland) as a standard.

#### Spectrophotometric assay

The assay mixture for NOX activity measurement contained 1 mol l^−1^ Tris–HCl buffer (pH 7.5), 1 mmol l^−1^ sodium 3, 3′-(−[(phenylamino)carbonyl]-3,4-tetrazolium)-bis(4-methoxy-6-nitro)benzene-sulfonic acid hydrate (XTT, Sigma-Aldrich, Poland), 1 mmol l^−1^ nicotinamide adenine dinucleotide phosphate hydrogen (NADPH, Sigma-Aldrich, Poland), and 20 μg of membrane proteins. XTT reduction by O_2_^−^ was determined at 492 nm (Able et al. 1998; Potocký et al. 2012). The rates of O_2_^−^ generation were calculated using an extinction coefficient of 2.16 × 10^4^ M^−1^ cm^−1^.

#### In-gel assay after native electrophoresis

Native polyacrylamide gel electrophoresis (PAGE) was carried out at 4 °C and 180 V in the Laemmli (1970) buffer system without sodium dodecyl sulfate (SDS). On each lane, 30 μg of proteins from the membrane fractions were loaded. NADPH-dependent O_2_^•−^-producing capabilities of the membrane fractions were assayed in gels by the blue formazan (NBT )reduction method described by Sagi and Fluhr (2001), with some modifications. The gels were incubated in the dark for 20 min in a reaction mixture containing 50 mmol l^−1^ Tris–HCl buffer (pH 7.5), 0.2 mmol l^−1^ NBT, 0.1 mmol l^−1^ MgCl_2_, and 1 mmol l^−1^ CaCl_2_. NADPH (0.2 mmol l^−1^) was added, and the appearance of blue bands was monitored. The reaction was stopped by the immersion of gels in distilled water. The gels were scanned in an Epson Perfection V700 Photo Scanner equipped with the Epson Scan program.

### Histochemical localization of O_2_˙ˉ and H_2_O_2_

Hypocotyls were infiltrated with a solution of 0.5 mg/ml NBT prepared in a 10 mmol l^−1^ potassium phosphate buffer, pH 7.2, or a solution of 2 mg ml^−1^ 3,3′-diaminobenzidine (DAB, Sigma-Aldrich, Poland) prepared in water, pH 5.5. Infiltration was carried for 1 h in the dark at room temperature. When the pale yellow NBT reacts with a superoxide, a dark blue insoluble formazan compound is produced (Fryer et al. 2002). DAB forms a deep brown polymerization product upon reaction with hydrogen peroxide in the presence of peroxidases (Thordal-Christensen et al. 1997). The staining solution was removed and samples incubated in NBT were rinsed with PBS and illuminated 150–200 mmol m^−2^ s^−1^ for 1 h (cool-fluorescent light) in order to make NBT staining more intense. Samples incubated in DAB were rinsed with distilled water. Chlorophyll was extracted with ethanol–chloroform (80/20 %; *v*/*v*) supplemented with 0.15 % (*v*/*v*) trichloroacetic acid. The bleached explants were submerged in a glycerin–water (1:1; *v*/*v*) solution and mounted on slides for light microscope (Nikon Eclipse E200) observations. Pictures were taken using a Canon EOS 450D device camera. For image storage and procession, Precoptic Co. Cool View software was employed.

### Statistical analysis

For each experiment, the means of three (in vitro culture, histochemical, and cytological studies) or five (biochemical analyses) replicates were calculated. The experiments were repeated three times. Statistical differences between means (*p* ≤ 0.05) were determined by two-way ANOVA followed by Tukey’s multiple range test using STATISTICA for Windows ver. 8.0 (StatSoft, Inc., Tulsa, OK, USA).

## Results

### Histological studies

**Fig. 1:**
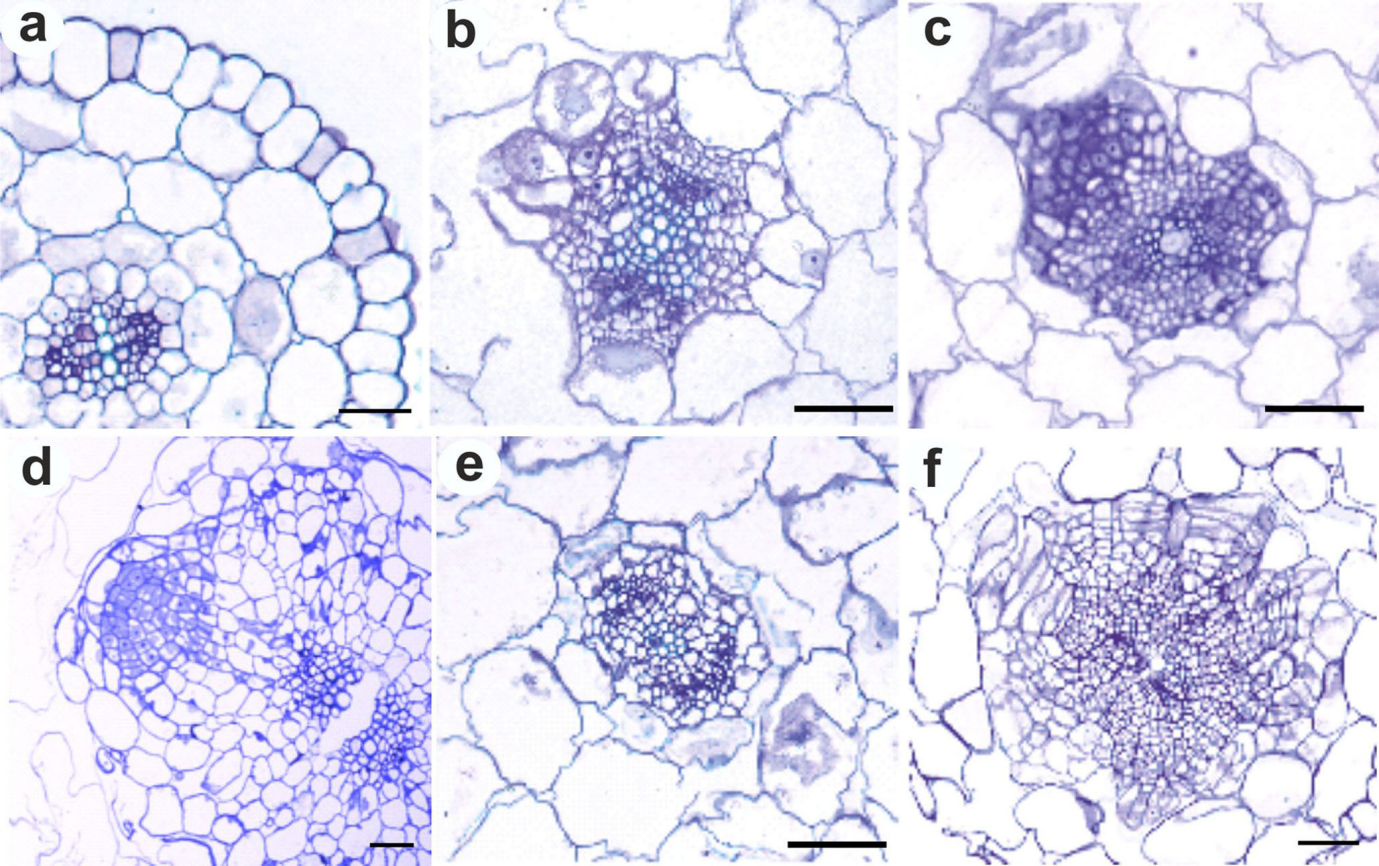
Toluidine blue staining of transverse section through **a** initial explant, **b** hypocotyl maintained on RIM for 3 days, **c** hypocotyl maintained on RIM for 5 days, **d** hypocotyls maintained on RIM for 7 days, **e** hypocotyls maintained on RIM + DPI for 3 days, and **f** hypocotyls maintained on RIM + DPI for 5 days. *Scale bar* = 50 μm

Transverse sections made through the hypocotyls before explantation showed presence of epidermis, few layers of cortex, and vascular cylinder of diarch structure. No cell division throughout hypocotyl explant was visible (Fig. 1a). Histological studies of hypocotyls at day 3 of culture on RIM showed intensive mitotic activity by parenchymatic cells within the vascular cylinder (Fig. 1b). Meristemoids were clearly visible in the outermost region of the vascular cylinder at day 5 of culture (Fig. 1c). Root primordia with apical meristems coated with root cap were regularly observed in explants maintained for 7 days on RIM (Fig. 1d). Hypocotyls developed roots as early as after 10 days of continuous culture on RIM supplemented with NAA. Roots were observed on almost all explants (90 %). In contrast, adding 0.1 μM DPI to the culture media inhibited root formation completely. Meristematic activity occurred only occasionally in cells from the vascular cylinder of hypocotyls at day 3 of culture on RIM + DPI (Fig. 1e). During prolonged culture, we observed some dividing cells in the inner cortex layer of the vascular cylinder (Fig. 1f). These cell divisions occurred in different planes and led to the formation of more or less compact groups of parenchyma-like cells.

### Endogenous concentration of H_2_O_2_

**Fig. 2:**
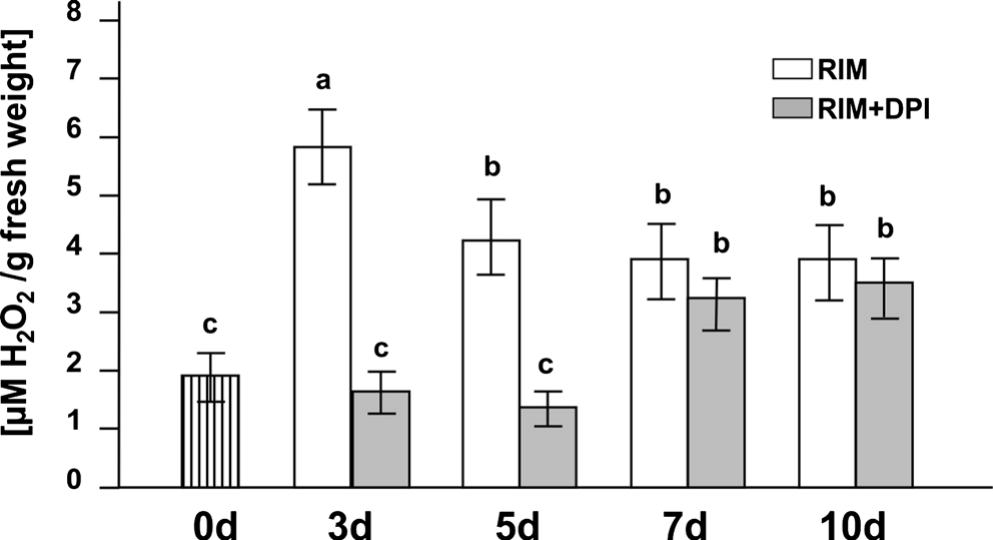
Endogenous level of H_2_O_2_ in initial explants and in hypocotyls cultured on RIM as well as on RIM + DPI. *Bars* represent the mean ± SD values (*n* = 5). Values sharing the same letter are not significantly different (*p* ≤ 0.05) according to Tukey’s multiple test

The H_2_O_2_ concentration strongly increased during the early stages of culture (3 days) (Fig. 2). Two days later (5 days), the hydrogen peroxide level decreased and remained unchanged at days 7 and 10 but still higher than in control material (0 day). Culture of hypocotyls on medium containing DPI (RIM + DPI) did not lead to changes in the hydrogen peroxide concentration in explants at days 3 and 5 versus the control material (0 day), but on days 7 and 10, the concentration increased to levels similar to those in hypocotyls cultured on RIM.

### Electron microscopy of H_2_O_2_ localization

**Fig. 3:**
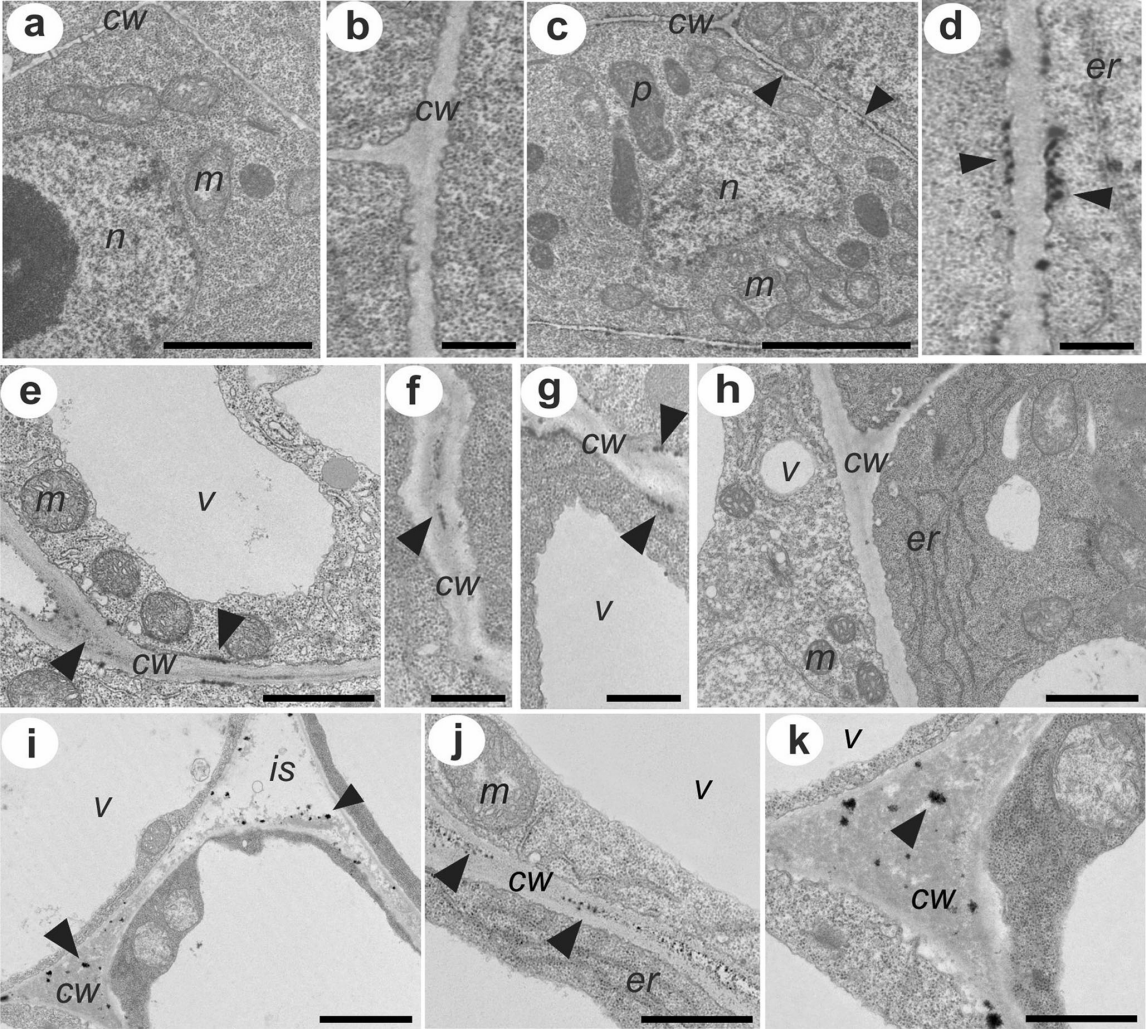
Ultrastructural localization of H_2_O_2_: **a**, **b** Cells from the vascular cylinder of hypocotyls cut from a seedling; **c**, **d** cells from the vascular cylinder of hypocotyls cultured for 3 days on RIM; **e**, **f**, **g** cortex cells from hypocotyls cultured for 3 days on RIM; **h** cells from the vascular cylinder of hypocotyls cultured for 3 days on RIM + DPI; **i**, **j**, **k** cortex cells from hypocotyls cultured for 3 days on RIM + DPI. *Arrowheads* point to cerium perhydroxide precipitates. *p* plastid, *cw* cell wall, *er* endoplasmic reticulum, *is* intercellular space, *n* nucleus, *v* vacuole, *m* mitochondrium. *Scale bar*: **a**, **c** = 2 μm; **b**, **d**, **f**, **g** = 0.5 μm; **e**, **h**, **i**, **j**, **k** = 1 μm

Electron-dense precipitates of cerium perhydroxides formed after the reaction of CeCl_3_ with endogenous H_2_O_2_ were not observed in the cells of hypocotyls before explantation (Fig. 3a, b). In contrast, in material from hypocotyls cultured for 3 days on RIM, we found cerium perhydroxide deposits in actively dividing cells within the vascular cylinder (Fig. 3c, d) as well as in the highly vacuolated cortex cells (Fig. 3e, f, g). Vascular cylinder cells and cortex cells differed in the distribution of cerium perhydroxide deposits. Actively dividing cells within the vascular cylinder showed the presence of hydrogen peroxide along the entire cell wall–plasma membrane interface (Fig. 3c, d), while cortex cells accumulated hydrogen peroxide not only at the cell wall–plasma membrane interface but also mostly inside their cell walls (Fig. 3e, f, g). In hypocotyls cultured for 3 days on RIM + DPI, vascular cylinder cells did not show electron-dense precipitates of cerium perhydroxides (Fig. 3h), and parenchymatic cortex cells had cerium perhydroxide precipitates in the cell walls (Fig. 3i, j, k) and mostly in the intercellular spaces between adjacent cells (Fig. 3k).

### NADPH oxidase activity

#### Spectrophotometric analysis

**Fig. 4:**
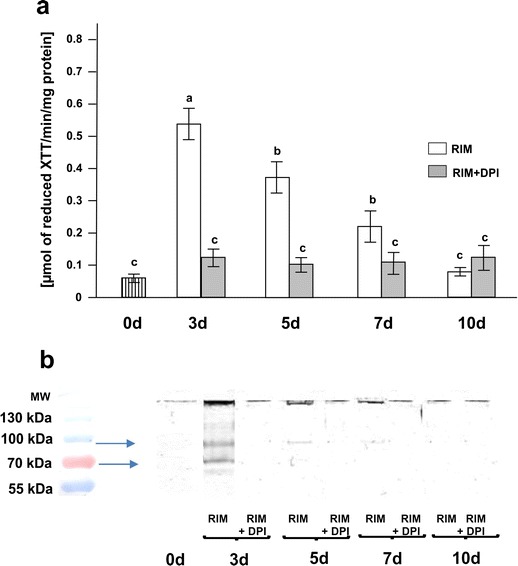
Studies on NOX activity: **a** spectrophotometric analysis of NOX activity in initial explants and in hypocotyls cultured on RIM as well as on RIM + DPI. *Bars* represent mean ± SD values (*n* = 5). Values sharing the same letter are not significantly different (*p* ≤ 0.05) according to Tukey’s multiple test. **b** Visualization of NOX isoforms on polyacrylamide gels after the activity staining of electrophoretically separated proteins from membrane fractions isolated from initial explants as well as from hypocotyls cultured on RIM and RIM + DPI. *MW* molecular weight marker (PageRuler Prestained Protein Ladder, Thermo Scientific)

Production of soluble yellow XTT formazan, formed due to reduction of XTT by O_2_^•−^ generated by NADPH oxidase (Potocký et al. 2012), was measured in plasma membrane fractions isolated from hypocotyls cultured on RIM and RIM + DPI. The concentration of XTT formazan was highest on day 3 of culture on RIM (Fig. 4a). Prolonged culture led to a gradual decrease in XTT formazan production. After 10 days of culture, the level of the XTT reduction product was comparable to that in control samples. DPI, an inhibitor of NOXs, significantly slowed the increase of O_2_^•−^ production; the concentration of XTT formazan was low and similar to that in control samples.

#### Visualization of NOX activity on polyacrylamide gels

The activity staining of gels after separation of the proteins from membrane fractions isolated from hypocotyls cultured for following days on RIM revealed the presence of two NADPH-dependent O_2_^•−^-producing bands corresponding to the activity of NOX isoforms with 100 and 70 kD molecular mass. They were most intense in samples from explants cultured for 3 days on RIM (Fig. 4b). After this time, the intensity of the detected bands decreased; only the band corresponding to 100 kDa molecular mass remained slightly active. No bands corresponding to NADPH oxidase isoforms activities were detected in the control material (0 day) nor in the samples from hypocotyls cultured on RIM + DPI.

### Histochemical localization of O_2_^•−^ and H_2_O_2_

**Fig. 5:**
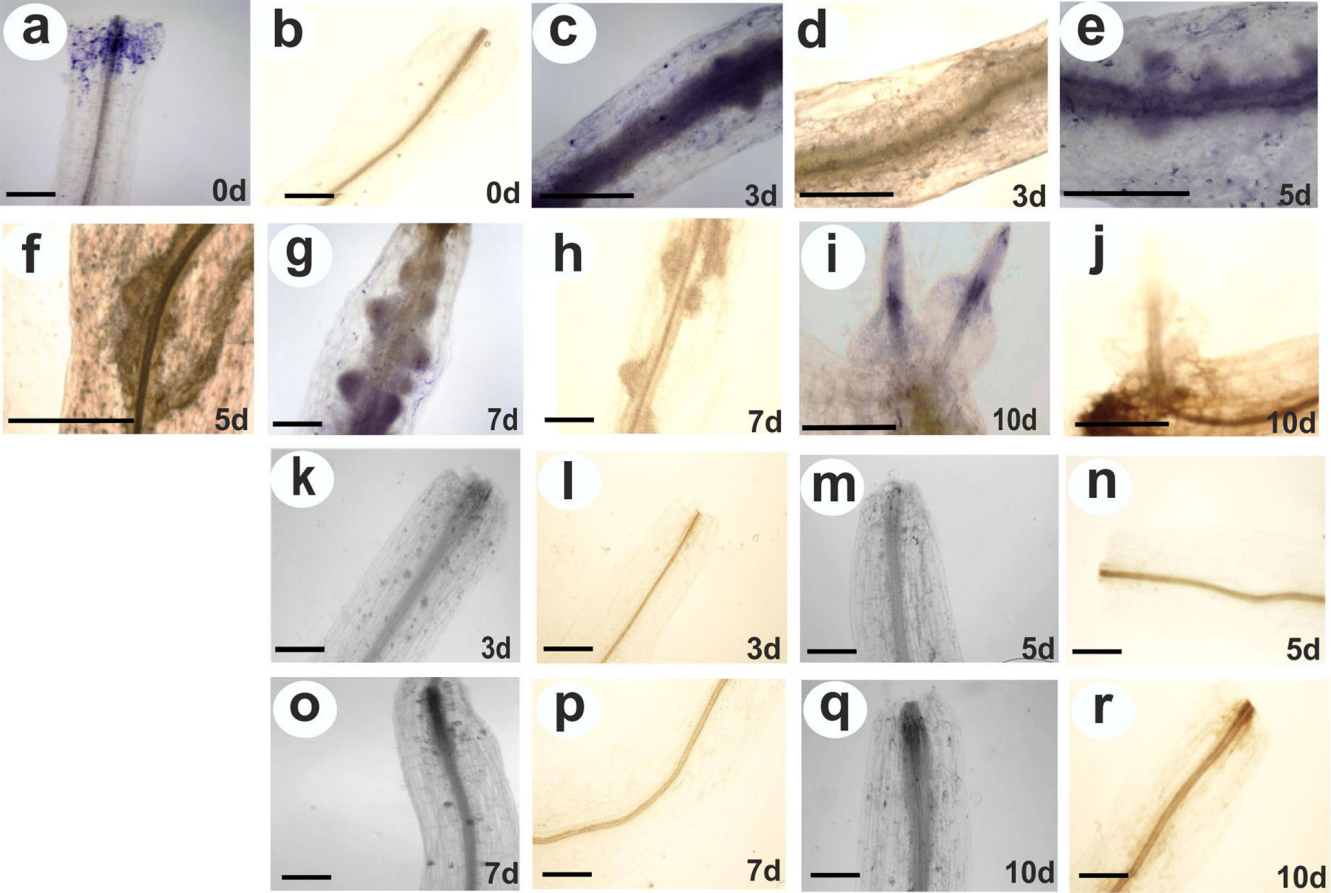
Visualization of ROS distribution after **a**, **c**, **e**, **g**, **i** NBT staining of O_2_^•−^ in initial explant (0 day) and hypocotyls during subsequent days of culture on RIM (3, 5, 7, 10 days); **b**, **d**, **f**, **h**, **j** DAB staining of H_2_O_2_ in initial explant (0 day) and hypocotyls during subsequent days of culture on RIM (3, 5, 7, 10 days); **k**, **m**, **o**, **q** NBT staining of O_2_^•−^ in hypocotyls during subsequent days of culture on RIM + DPI (3, 5, 7, 10 days); **l**, **n**, **p**, **r** DAB staining of H_2_O_2_ in hypocotyls during subsequent days of culture on RIM + DPI (3, 5, 7, 10 days). *Scale bar* = 1 mm

Hypocotyls excised from seedlings (0 day) had insoluble blue formazan precipitates in epidermis cells at the cut ends, indicating strong O_2_^•−^ accumulation (Fig. 5a). In the vascular tissue of hypocotyls at the time of excision (0 day), we observed dark brown staining, uniformly faint, resulting from DAB polymerization due to the presence of H_2_O_2_ (Fig. 5b).

After 3 days of culture on RIM, the blue formazan precipitates were strongly accumulated in vascular bundle cells (Fig. 5c). At the same time, DAB precipitates were also strongly accumulated in vascular cylinder cells as well as in cortex cells (Fig. 5d). At day 5 of culture, superoxide radicals and hydrogen peroxide were disclosed in meristemoids formed inside the hypocotyl stele, and H_2_O_2_ was visualized in cortex cells (Fig. 5e, f). After 7 days of explantation, a high concentration of blue formazan precipitates appeared at the tip of the primordia. At the same time, clearly visible root initials were strongly and evenly stained with DAB (Fig. 5h). At day 10, the accumulation of superoxide radical anions was limited to the tip and middle part of growing roots (Fig. 5i). DAB staining was less intense in newly developed roots, and the presence of hydrogen peroxide was indicated in their vascular tissue (Fig. 5j).

At days 3 and 5, hypocotyls cultured on RIM + DPI did not exhibit rhizogenic potential and NBT staining did not show high accumulation of superoxide radicals (Fig. 5k, m). Some blue formazan precipitates were visible in epidermis cells. The concentration of blue formazan precipitates was higher at days 7 and 10 at the cut ends, but staining intensity was lower than in hypocotyls cultured on RIM at the same days (Fig. 5o, q). DAB staining disclosed the highest concentration of hydrogen peroxide in the vascular tissue, but it was not visualized in other cells of the vascular bundle and cortex (Fig. 5l, n, p, r).

## Discussion

In other recent work, hypocotyls of *M. crystallinum* cultured on a medium supplemented with 2,4-dichlorophenoxy-acetic acid (2,4-D) exhibited high rhizogenic potential and induction of root formation was found to depend on high endogenous H_2_O_2_ and the activity of specific isoforms of some antioxidant enzymes (Konieczny et al. 2014). Here, we show that another synthetic auxin, NAA, influences rhizogenesis similarly. Auxins play a central role in the control of cell and plant growth, but oxidative stress caused by overproduction of ROS also affects growth and development. Pasternak et al. (2005) showed that oxidative stress initiates cell division and the formation of morphogenic cell clusters in the pericycle area of *Arabidopsis* seedlings but that in the absence of externally sourced auxin the oxidative stress had no effect on morphogenesis induction. They concluded that the effect of oxidative stress was to enhance an auxin-driven process, leading to morphogenesis. Similarly, our results indicated that not auxin alone but rather auxin accompanied by an appropriate level of H_2_O_2_ is necessary for rhizogenesis from *M. crystallinum* hypocotyls cultured on medium containing NAA. When we manipulated the level of ROS by adding DPI—an inhibitor of NOXs to the rhizogenesis-inducing medium—meristematic activity decreased within vascular cylinder cells and the formation of root primordia was inhibited. Konieczny et al. (2014) observed a similar effect after applying ascorbate (AA) to rhizogenic medium containing 2,4-D. It led to decreased hydrogen peroxide levels in the explants and blocked the rhizogenic potential. NOXs are an integral membrane protein complex known to be the main source of ROS present in the apoplast and playing roles in cell defense, development, and redox-dependent signaling (Fluhr 2009). AA is by far the most abundant low-molecular-weight antioxidant in the apoplast; its key function is redox buffering in the apoplast, which protects the plasmalemma from oxidative damage (Pignocchi and Foyer 2003). Both of those findings point to the specific role of apoplast redox status in induction of morphogenic responses in cultured explants.

Production of ROS in the apoplast can drive the oxidative reactions towards loosening of cell walls in growing tissues (Fry 1998; Potikha et al. 1999; Liszkay et al. 2004) and can stiffen cell walls as growth ceases and cells differentiate (Hohl et al. 1995; Brisson et al. 1994; Schopfer 1996; Ros-Barcelo et al. 2002; Foreman et al. 2003). Our measurements of endogenous H_2_O_2_ during successive stages of rhizogenesis from hypocotyls cultured on RIM indicated a significant increase of H_2_O_2_ after 3 days of explantation, correlated with induction of mitotic activity in cells within the vascular cylinder (Figs. 1b and 2). Ultrastructural observations showed electron-dense deposits of cerium perhydroxides, indicating the presence of H_2_O_2_ in the cell wall–plasma membrane interface of actively dividing cells from the vascular cylinder of hypocotyls cultured for 3 days on RIM (Fig. 3c, d). Cerium perhydroxide precipitates were also observed in cortex cells after 3 days of hypocotyl culture on RIM as well as on RIM with DPI added. In these cells, however, H_2_O_2_ was localized in the cell walls, especially in the middle lamella of cell walls between adjacent cortex cells (Fig. 3 e, f, i, j, k). The differences in localization of cerium perhydroxide precipitates between vascular cylinder cells and cortex cells probably are due to differences in the function of ROS in those cells and the differential activity of various enzymes regulating the ROS level. We postulate that NADPH oxidase is responsible for H_2_O_2_ level regulation in vascular cylinder cells, while cell wall-bound enzymes, most probably peroxidases (POD), participate in control of H_2_O_2_ content in cortex cells. ROS produced due to NOX activity have been implicated in regulation of cell cycle progress, organization of microtubule arrays, nuclear envelope dynamics, and cell plate formation (Livanos et al. 2012). Our results on NADPH oxidase activity during successive stages of rhizogenesis give evidence that NOX functions as the main source of ROS in actively dividing cells, since its activity increased significantly in explants cultured for 3 days (Fig. 4a) and was correlated with the increase in endogenous hydrogen peroxide (Fig. 2). Moreover, adding DPI led to decreases of NADPH oxidase and H_2_O_2_ in cultured explants (Figs. 3a and 2). The spectrophotometrically measured activity of NADPH oxidase represented the sum of two isoforms visualized after activity staining of membrane proteins separated after electrophoresis (Fig. 4b). Bands corresponding to NADPH oxidase isoforms were present at masses of 100 and 80 kDa, similar to the bands of NOX isoforms visualized after protein separation from cells of other plant species (Sagi and Fluhr 2001). Plant NOXs, similarly as mammals NADPH oxidases, consist of glycoprotein gp91^phox^ (phox for phagocyte oxidase), which conserved all the catalytic machinery (Hervé et al. 2006). A number of homologues of gp91^phox^ named respiratory burst oxidase homologues have been discovered in higher plants. *Arabidopsis thaliana* contains at least ten gp91^phox^ homoloques designated as *Atrboh A-J* (*A. thaliana* respiratory burst oxidase homologues). Transcripts of *Atrboh* are also known tissue-specifically distributed: for *AtrbohD* and *F*, the transcripts are mainly expressed in the roots (Sagi and Fluhr 2006). On the basis of this information, we can speculate that the visualized bands might correspond with NOX isoforms engaged in root development; however, to describe the real nature of the visualized proteins, further studies must be performed.

Cerium perhydroxide precipitates were not visible in vascular bundle cells of DPI-treated hypocotyls but were still present in cortex cells of explants from the DPI treatment, as in the cortex cells of those cultured on RIM alone. This suggests that a regulatory compound other than NOX influences the hydrogen peroxide level in the cortex cells. POD activity might be responsible for the presence of H_2_O_2_ detected as cerium perhydroxide precipitates in the walls of cortex cells. Cortex cells from hypocotyls cultured for 3 days on RIM and on RIM with DPI followed developmental fates different from those of vascular cylinder cells; they responded to auxin by elongation growth. They were also exposed to the pressure of proliferating cells from the vascular cylinder, so the walls of cortex cells had to be modified by loosening and stiffening reactions. In our previous work, POD activity increased in hypocotyls after explantation and was significantly higher during new root development (Konieczny et al. 2014). PODs are considered to be bifunctional enzymes (Passardi et al. 2004). Various isoforms of POD are present in plant cell walls (Kukavica et al. 2009), where they control cell wall elasticity. Dunand et al. (2007) showed that POD in the presence of O_2_^•−^ should produce the OH^•^ needed for cell wall loosening, which facilitates cell division and elongation; conversely, the presence of H_2_O_2_ and POD drives cell wall stiffening via crosslinking of structural proteins in the wall.

The dual nature of peroxidases was also reflected in the histochemical localization of O_2_^•−^ and H_2_O_2_. In our study, differences in the distribution of superoxide radicals and hydrogen peroxide appeared already in the initial explants. We observed a high accumulation of blue formazan precipitates, indicating a high concentration of superoxide radicals, at both ends of hypocotyls excised from seedlings (Fig. 5a). At the same time, at those cut ends, we did not note high accumulation of the DAB precipitates disclosing hydrogen peroxide production (Fig. 5b). It is known that just after wounding, plants transiently produce ROS; they take part in the production of various compounds forming a physical barrier at the wound site and they also help activate a systemic response that relies on cell-to-cell signal transduction. Production of superoxide radicals during wounding is at maximum several minutes after injury, while the first increase in hydrogen peroxide production occurs after several hours (León et al. 2001). This might explain the differences in the visualization of these molecules in the initial explants. Our study produced evidence that O_2_^•−^ and H_2_O_2_ have their own distinct accumulation zones during the stages of rhizogenesis and thus might have different roles in the process of root formation from hypocotyls cultured in vitro. During early stages of rhizogenesis, characterized by high cell division activity and the formation of meristemoids within the hypocotyl stele, we noted intense NBT staining of superoxide radicals in vascular cylinder cells (Fig. 5c, e). Hydrogen peroxide was indicated there but was also disclosed in cortex cells (Fig. 5d, f). Visualization of superoxide radicals during root development in the tip of the root primordia, at the tip of growing roots, and in the middle part of developing organs, might correspond to the formation of quiescent centers (QC). They function as zones of initial cells for root apical meristems (Jiang and Zhang 2002). The middle parts of growing roots, exhibiting strong superoxide anion accumulation, could represent the elongation zone of roots. The presence of a high level of O_2_^•−^ in this area is a common feature of seed plants, possibly related to promotion of increased cell wall extensibility within this part of the root (Liszkay et al. 2004). From our results, it can be concluded that the superoxide radicals are engaged in processes of rhizogenesis induction that rely on induction of competent cell division, while hydrogen peroxide is engaged in developmental processes relying mainly on cell growth. From a work by Bao et al. (2009), we know that the major regulatory events during plant organogenesis occur in early stages of this process induction, before visible changes in explant morphology become obvious. ROS detection during early phases of rhizogenesis might be usefully exploited for morphogenesis in in vitro culture systems as ROS are primary markers of the capacity of differentiated cells to induce regeneration.
